# Size matters: Natural experiments suggest the dear enemy effect is moderated by pack size in African wild dogs

**DOI:** 10.1002/ece3.11298

**Published:** 2024-04-17

**Authors:** Megan J. Claase, Mike I. Cherry, J. Weldon McNutt, Peter J. Apps, Neil R. Jordan

**Affiliations:** ^1^ Department of Botany & Zoology Stellenbosch University Matieland South Africa; ^2^ Botswana Predator Conservation, Wild Entrust Africa Maun Botswana; ^3^ Department of Zoology Nelson Mandela University Port Elizabeth South Africa; ^4^ Centre for Ecosystem Science, School of Biological, Earth and Environmental Sciences University of New South Wales (UNSW) Sydney New South Wales Australia; ^5^ Taronga Institute of Science and Learning, Taronga Western Plains Zoo, Taronga Conservation Society Dubbo New South Wales Australia

**Keywords:** camera‐trap, carnivore, *Lycaon*, scent marking, social behaviour, territoriality

## Abstract

Remote monitoring of communal marking sites, or latrines, provides a unique opportunity to observe undisturbed scent marking behaviour of African wild dogs (*Lycaon pictus*). We used remote camera trap observations in a natural experiment to test behavioural scent mark responses to rivals (either familiar neighbours or unfamiliar strangers), to determine whether wild dogs exhibit the “dear enemy” or “nasty neighbour” response. Given that larger groups of wild dogs represent a greater threat to smaller groups, including for established residents, we predicted that the overarching categories “dear enemy” vs. “nasty neighbour” may be confounded by varying social statuses that exists between individual dyads interacting. Using the number of overmarks as a metric, results revealed an interaction between sender and receiver group size irrespective of familiarity consistent with this prediction: in general, individuals from large resident packs overmarked large groups more than they overmarked smaller groups, whereas individuals from smaller packs avoided overmarking larger groups, possibly to avoid detection. Monitoring a natural system highlights variables such as pack size that may be either overlooked or controlled during scent presentation experiments, influencing our ability to gain insights into the factors determining territorial responses to rivals.

## Graphical abstract

Using camera traps to generate behavioural data to act as a “natural experiment” to determine African wild dog responses to rivals (unfamiliar strangers vs. familiar neighbours). Authors found that group size impacts behavioural responses, and highlights shortcomings of scent presentation experiments in determining territorial responses to rivals.

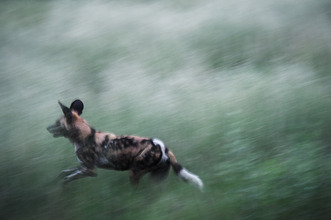

## 1 INTRODUCTION

Individuals and social groups from many species inhabit discrete, relatively fixed territories that are defended against conspecifics, and contain limiting resources such as feeding sites, breeding sites, or mates (Maher & Lott, 1995). Territorial maintenance and defence is costly and can be achieved actively (through physical combat) or passively (through auditory, visual or olfactory signals) (Gese, 2001), with scent marks often playing a key role in the latter (Johnson, 1973). In canids, territoriality is widespread, and defence often occurs passively through vocalisations and scent marking (e.g. grey wolves, *Canis lupus*, Harrington, 1987; Harrington & Mech, 1979, 1983; Peters & Mech, 1975; Rothman & Mech, 1979; Ethiopian wolves, *Canis simensis*, Sillero‐Zubiri & Macdonald, 1998; coyotes, *Canis latrans*, Bowen & Cowan, 1980; Camenzind, 1987; Gese, 2001; Gese & Ruff, 1997; and African wild dogs, *Lycaon pictus*, Apps et al., 2022; Claase et al., 2022; Claase et al., 2024; Parker, 2010). Scent marking studies in the wild have focused primarily on the scent marking behaviour of the individuals who send a scent signal, much less information has been gathered on the behaviour of receivers of a signal, as there are often considerable delays between signals being sent and received, which limits our understanding of the role of scent in territoriality.

Observations of direct encounters (Gosling, 1982) between conspecifics are rare among carnivores (Bekoff & Wells, 1986; Camenzind, 1987; Jordan et al., 2017; Mech, 1993, 1994), and signal‐based presentation experiments therefore provide a method to gauge territorial responses of resident individuals to simulated intrusions by conspecifics. A documented way to minimise energetic costs associated with territoriality is for residents to tailor their responses depending on the threat that intruders represent (Fisher, 1954). Two categories of rival are commonly identified: neighbours and non‐neighbours (termed “strangers”). In many species, residents often respond less aggressively to neighbours than to strangers (see Temeles, 1994; Ydenberg et al., 1988), termed “dear enemy” (sensu Fisher, 1954; Temeles, 1994), which can be explained by two hypotheses: the familiarity hypothesis (Ydenberg et al., 1988) and the threat level hypothesis (Temeles, 1994). The familiarity hypothesis argues that there is a lower likelihood of conflict from a neighbour (a familiar) compared to a stranger (an unfamiliar), for example due to the lower likelihood of role mistakes in territorial contests (Ydenberg et al., 1988), which enables relationships between familiar neighbours to settle, allowing the conservation of time and energy, and reducing the risk of injury (Wilson, 1975). The threat level hypothesis argues that due to the differences in resources required by strangers and neighbours, strangers pose a greater threat to residents (Temeles, 1994), and may seek to acquire territory in a more spatially and temporally unpredictable way (Jordan et al., 2007) than do neighbours.

While data from many animals support the dear enemy hypothesis (e.g. Nearctic river otters, *Lontra canadensis* Oldham & Black, 2009; aardwolf, *Proteles cristatus* Sliwa, 1996), some species respond more aggressively to neighbours than to strangers (e.g. banded mongoose, *Mungos mungo*, Manser & Müller, 2007; striped mice, *Rhabdomys pumilio*, Schradin et al., 2010; chimpanzees, *Pan troglodytes*, Herbinger et al., 2009). It has been suggested that this so‐called “nasty neighbour” effect is more common in social species (Christensen & Radford, 2018), and tends to occur when neighbours represent a greater threat to residents than do strangers, which can happen when group sizes differ; for example, dispersing groups may be smaller due to having only recently formed and therefore these “strangers” would pose less of a threat than established neighbouring groups. Indeed, the threat level hypothesis predicts a stronger response to larger than smaller groups (see Manser & Müller, 2007). The effect of group size of the individuals which sent the signal (the “senders”) on scent marking responses by the individuals which receive a scent signal (the “receivers”) has not been investigated, and sender group size is often held constant in experiments (e.g. banded mongoose, Manser & Müller, 2007). Given that larger groups are more successful in some species (e.g. meerkats, *Suricata suricatta*, Clutton‐Brock et al., 1999), larger groups may advertise their territory more boldly by scent marking more. Additional nuance exists in some social groups, where individuals (or categories of individuals) vary in the cost/benefits of interacting with individuals from other social groups. Consequently, responses to intruders may vary within groups according to an individual's sex, social status and dispersal likelihood, as well as the resident's access to resources within the group (Christensen & Radford, 2018).

Experiments simulating intrusions by neighbours and strangers demonstrate a range of responses in group living carnivores (e.g. banded mongoose, Jordan et al., 2010; Manser & Müller, 2007; dwarf mongoose, *Helogale parvula*, Christensen et al., 2016; Eurasian beaver, *Castor fibre*, Rosell & Bjørkøyli, 2002; dholes, *Cuon alpinus*, Ghaskadbi et al., 2022; African wild dogs, Parker, 2010). While these experiments provide insights into the responses to different stimuli, physical presentation of scents may be out of context spatially, temporally and socially, meaning that the experiments may not accurately reflect how group living species would respond under natural circumstances. In contrast to experiments, observations of undisturbed marking behaviour represent an ideal and underutilised method to study behavioural responses of groups and individuals within the neighbour‐stranger dyad.

Latrines are discrete locations where multiple individuals or groups come to deposit urine, faeces and other bodily secretions (Brown & Macdonald, 1985), and these sites have often been linked to a role in territorial defence (e.g. Ethiopian wolves, Sillero‐Zubiri & Macdonald, 1998; spotted hyaena, *Crocuta crocuta*, Henschel & Skinner, 1991; Mills & Gorman, 1987; brown hyaena, *Parahyaena brunnea*, Gorman & Mills, 1984). Remote monitoring of these long‐term shared marking sites offers an unparalleled and previously underexplored research opportunity to investigate territorial behaviour in a natural environment (although see Allen et al., 2014; King et al., 2017; Li et al., 2013).

African wild dogs (hereafter wild dogs) are wide ranging highly social large carnivores that use latrines, termed marking sites, to advertise territorial residence (Apps et al., 2022; Claase et al., 2022). Typically, only the dominant individuals breed within each pack, with subdominant individuals helping to raise offspring (Creel & Creel, 2002). When offspring reach 2–3 years of age, many disperse in single sex coalitions to seek mates, sometimes travelling large distances to form new reproductive groups with unrelated opposite sex individuals, or usurping and expelling same‐sex residents from an established pack (Behr et al., 2020; Cozzi et al., 2020; McNutt, 1996), creating intense same‐sex competition between resident dominant individuals and dispersers. Typically, dominant individuals scent mark more than subdominants, with dominant females scent marking more than dominant males in the intra‐pack context (Claase et al., 2024; Jordan et al., 2014). Both resident packs and dispersers scent mark at shared marking sites (Claase et al., 2022), which creates a unique opportunity in this species to monitor the behaviour of dispersers (strangers), and the reaction of residents to signals from strangers and neighbours at the same sites.

Previous experimental scent presentations to wild dogs have shown that dominant individuals scent mark more than subdominants and that they respond to stranger scent more than to neighbour scent (Parker, 2010), results that were paralleled recently in Asiatic wild dogs or dholes (Ghaskadbi et al., 2022). Consequently, we would expect wild dogs to exhibit this “dear enemy” response under natural conditions at shared marking sites. Specifically, we predict that resident packs would scent mark more in response to dispersing coalitions (strangers) than to resident packs (neighbours). We would also expect pack size to influence the likelihood of scent marking, with larger packs being “bolder” and scent marking stranger scent more than smaller packs. Furthermore, if strangers present a greater threat than neighbours, we would expect individuals within resident packs to overmark same sex dispersers more than same sex neighbours. Finally, we would predict scent marking patterns in the inter‐pack context to be similar to those recorded in this species in the intra‐pack context, namely that dominants scent mark more than subdominants, and that dominant females scent mark most.

## 2 METHODS

### 2.1 Study site and population

Data were collected between January 2019 and December 2020 on a free‐ranging subpopulation of African wild dogs on the eastern fringes of the Okavango Delta in northern Botswana (approx. 2600 km^2^ centred on 19°31′ S, 23°37′ E), which have been studied since 1989 by Botswana Predator Conservation (BPC). Data were collected from 11 contiguous resident packs and 13 dispersing coalitions. A *pack* is a stable group containing at least one adult male and one unrelated adult female (Creel & Creel, 2002), while a *disperser* is an individual or a same‐sex group of individuals that are not part of a pack (Creel & Creel, 2002). In this study, a *group* refers to both packs and dispersing coalitions. All individuals were identified by their unique pelage patterns, tail stripes and ear notches, with 76% (63/83) of the individuals in this study of known age and origin. Any individuals not born into the study population (typically dispersers) were aged according to size, pelage and tooth wear (if captured for collaring). Dominance was identified through attributes such as tandem overmarking (see Jordan et al., 2013), mate guarding and mating, and resting together. Additionally dominants generally eat first at kills, with the exception of the individual that made the kill and the young pups (Jordan et al., 2022), and dominants are usually the focus of pre‐departure rallies, a highly social greeting ceremony (Robbins, 2000).

### 2.2 Marking site monitoring and data processing

Behavioural data were collected over 24 months from 35 camera traps set up to record video at 21 shared marking sites in boundary or overlap zones between neighbouring resident packs (mean study days per SMS ± SD = 531.6 ± 139.1, range 257–730 days). Marking sites were initially located by following radio‐collared wild dogs and observing where they scent marked, with a location confirmed as a marking site when repeated visits were captured by camera traps (see Claase et al., 2022 for details). When wild dogs are not present and actively marking, their latrines are difficult to see except at the end of the dry season when scats may be visible, so it is likely that some marking sites were not detected by observers. Once identified, marking sites were equipped with camera traps (either Reconyx Ultrafire or Browning Strikeforce Pro XD) set to record 20 or 30s videos and aimed so that the field of view captured as much of the site as possible. Camera traps were housed in custom‐build metal boxes to protect them from wildlife and were mounted on 1.5 m metal poles. Marking sites were monitored continuously over the study period, except when cameras malfunctioned or were knocked over by wildlife. Cameras were routinely checked every 7–10 days, with SD cards and batteries changed as necessary. SD cards were backed up, and videos archived and classified according to species detected. For videos containing wild dogs; date, time, marking site *location* (a unique identifier for each marking site), camera number, pack identity, and *individual* dog identities were recorded in a spreadsheet alongside detailed behavioural observations, following methodology described elsewhere (see Claase et al., 2024). Each individual wild dog was identified, and their behaviours tracked and recorded in sequence, including *sniffing*, and scent marking (*urinating*, *defecating*, *rub‐rolling* and *dragging*). Rub‐rolling refers to an individual rolling on the ground; dragging refers to an individual dragging its hindquarters along the ground. Each scent‐mark series (one or more scent mark at a specific spot within a marking site) was given an identifying number (*Series ID*). Series were located in the videos using distance to obvious vegetation and landscape features. Behaviours by subsequent visitors to each series were recorded. Any sniffing events directed at a series with no prior or subsequent recorded scent‐marks were discarded in these analyses as they were likely not involved with observable communication events. All other sniffs were included in analyses with no temporal restrictions. Overmarking occurred when an individual sniffed a series and placed a scent‐mark over an existing scent‐mark (i.e. on the same series), such that the two marks overlapped (sensu Johnston et al., 1994). All first recorded marks at a series were discarded from analyses (*N* = 276) as it is impossible to determine whether it was the first mark in a series, or a mark in response to a pre‐existing mark preceding camera set up. A behavioural *session* was defined as the period during which a single group of wild dogs visited a single marking site. Only individuals that sniffed or scent‐marked were included in analyses (see Claase et al., 2024).

### 2.3 Statistical analysis

All statistical tests were conducted in R version 4.2.1 (R Development Core team, 2022) using *lme4* (Bates et al., 2015) and *glmmTMB* (Brooks et al., 2017), and analyses were restricted to inter‐group overmarking. To fully explore behavioural responses to rivals, we ran two analyses; the first from the perspective of resident packs (a group behaviour), and the second from the perspective of individuals within those resident packs (individual behaviours).

In the first analysis, to determine whether resident packs responded more strongly as a group to strangers than to neighbours, we ran a generalised linear mixed effect model (GLMM) with a Poisson error distribution looking at the factors that affected the total number of overmarks deposited during a session by all individuals, regardless of their sex or status. We included a variable *Previous Group Type*, which described the type of the group (stranger or neighbour to the resident) to visit a marking site immediately prior to the visit by the receiving pack, *Previous Group Size* (the number of adults >12 months in the group that visited the marking site immediately prior to the visit by the receiving pack), and *Pack Size* of the receiving pack as explanatory variables. Marking site *location* and *group ID* (packs and dispersers) were included as random terms to account for repeated measures. We used Akaike's information criterion (AICc, Akaike, 1974) to select the most parsimonious model (Harrison et al., 2018) from candidate models (Burnham & Anderson, 2004), which used all combinations of fixed effects and their interactions.

In the second analysis, we ran a GLMM with a binomial error distribution (1 = overmarking, 0 = no overmarking) to investigate the behavioural responses of individuals given assumed potential asymmetries in the costs and benefits of encountering neighbours and strangers. Specifically, we investigated if responses were different to neighbours vs. strangers and whether this varied by the dominance structure and sex of senders and receivers. We included *Sender Group Type*, which described the group type of the individual who sent the signal, with the individual either belonging to a “Stranger” group or a “Neighbour” group, *Sender Group Size*, *Pack Size* of the receiving pack, *Status* (of the receiver; “Dominant” or “Subdominant”), *Sex* (of the receiver; “M” male or “F” female), *Sender Sex* (“M” male or “F” female) and *Season* (“Mating” and “Non‐mating”; annual mating season March/April in this population) as explanatory variables. We included marking site *location*, *group ID* (packs and dispersers), and *individual* dog IDs as random terms to control for repeated measures. We used Akaike weights to determine the relative importance of each model (Grueber et al., 2011) and selected the most parsimonious model from candidate models including all explanatory variables and their biologically plausible two‐way interactions (Harrison et al., 2018).

### 2.4 Ethical note

Ethics approval was provided by UNSW Animal Ethics Committee (Approval number: 20/166B; application title: Large carnivore ecology and conservation research (Botswana); Chief Investigator: Dr. Neil Jordan). No animals were collared specifically for this study as data were gathered from an already established study population. Camera traps were used to monitor marking sites with videos set to use infra‐red at night to minimise disturbance to all animals. No behavioural changes were recorded among wild dogs, with camera traps being chiefly ignored (any investigation was by curious young individuals). Camera traps caused no harm or disturbance to any other animal species, although some camera traps were disturbed or knocked over by elephants or hyaenas.

## 3 RESULTS

**FIGURE 1:**
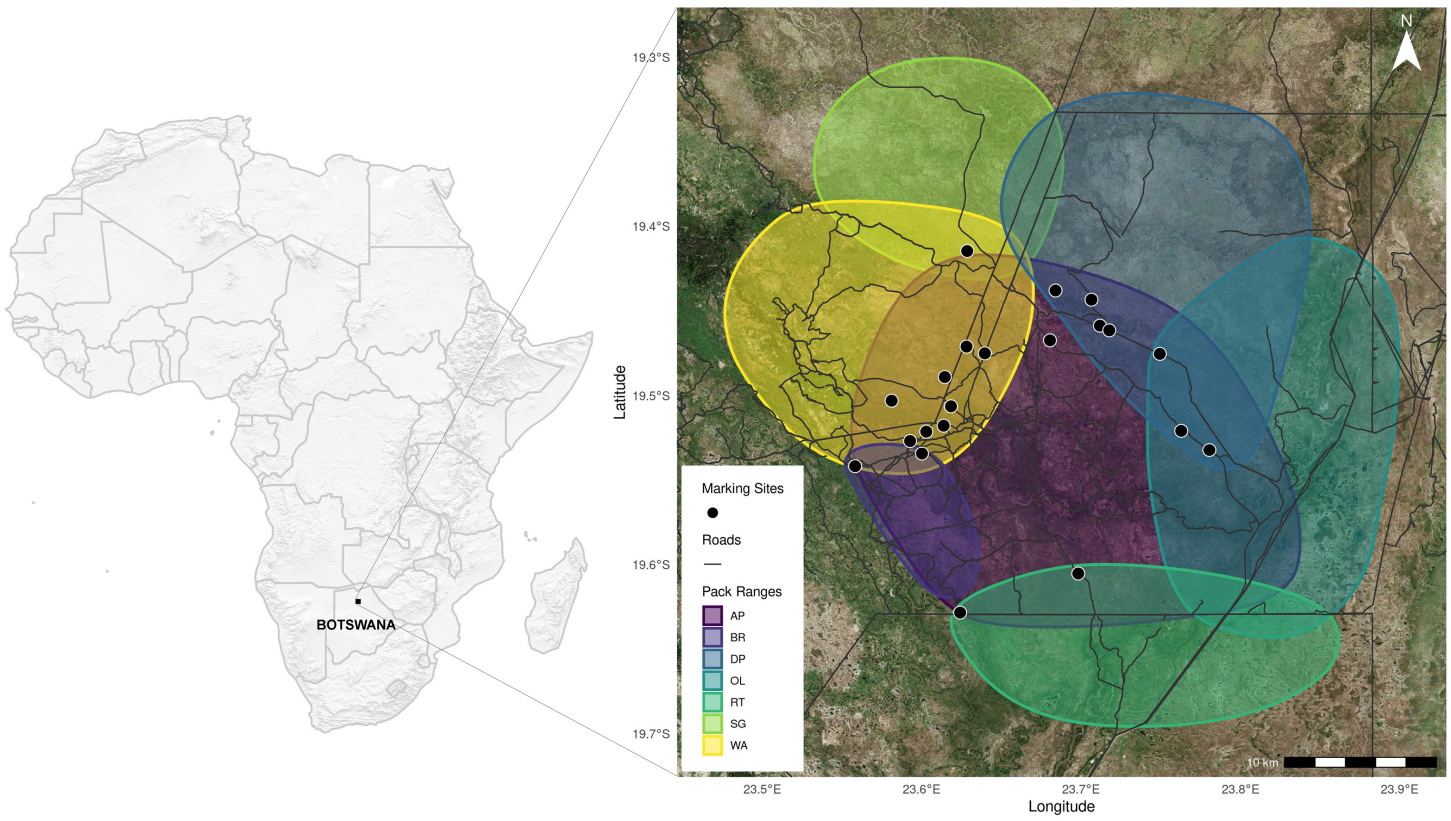
Estimated territories of African wild dogs in 2020 and their relation to marking sites in boundary or overlap zones. Territories are estimates of use based on sightings data, and are not hard boundaries. Seven of the eleven recorded packs are represented here at this point in time as packs and their territories changed over the 2‐year study period. Each marking site was visited by multiple packs and dispersing coalitions over the course of the study.

Eleven resident packs (mean pack size 12; range 2–19) responded to neighbour and stranger scent marks at 21 marking sites during 373 sessions over 24 months (Figure 1). Of these sessions, 281 involved scent communication between 83 individuals across 330 series (i.e. scent marks which were sniffed (*N* = 2577) by a member of a resident pack and either overmarked (*N* = 468) or not (sniffing events per series: 7.03 ± 4.95; marking events per series: mean ± SD = 2.38 ± 0.99)).

**FIGURE 2:**
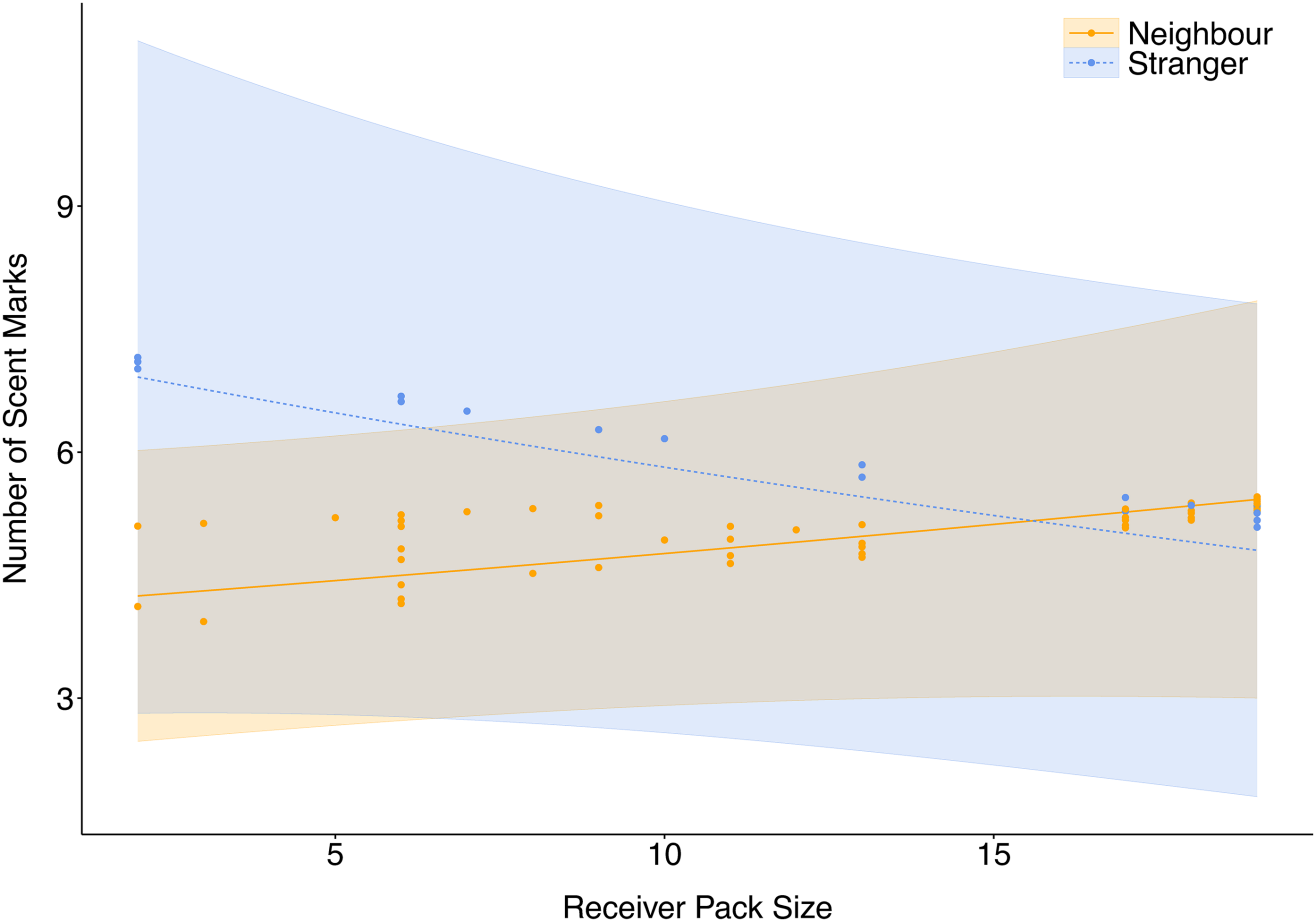
Predicted (line) generated from a GLMM and actual number (points) of scent marks deposited by wild dogs (Table 2.) investigating factors that affect the number of scent marks deposited by packs scent marking at a shared marking site. For visualisation purposes, previous group size was fixed at the mean. Data were drawn from 373 sessions from 11 resident packs across 21 marking sites.

For the first analysis, all candidate models were similar (within <2 AICc of the top performing model), with an AICc difference of only 0.1 between the two top performing models (Table 1). The most parsimonious model included the fixed effects pack_size, previous_group_size and previous_group_type, as well as the interaction between pack size and previous group type (Table 2). Overall wild dog packs were more likely to scent mark when the previous visit to a marking site was by a stranger group rather than a neighbouring group, thereby exhibiting a “dear enemy” response (Figure 2). At a finer scale, smaller packs scent marked more in response to strangers, and larger packs scent marked more in response to neighbours. All other interactions were excluded from the final model.

**TABLE 1:** Generalised linear mixed‐effect models (GLMMs) with a Poisson error distribution exploring how various factors affect the number of scent marks deposited by packs of wild dogs at shared marking sites.

m	Description	Df	logLik	AICc	Delta	Weight
Null				3092.8		
1	Pack_size + previous_group_size + previous_group_type + pack_size:previous_group_type	7	−1537.756	3089.8	0.00	0.206
2	Pack_size + previous_group_size + previous_group_type + pack_size:previous_group_size + pack_size:previous_group_type	8	−1536.770	3089.9	0.12	0.195
3	Pack_size + previous_group_size + previous_group_type + pack_size:previous_group_type + previous_group_size:previous_group_type	8	−1536.789	3090.0	0.15	0.191
4	Pack_size + previous_group_size + previous_group_type + pack_size:previous_group_size + pack_size:previous_group_type + previous_group_size:previous_group_type	9	−1536.230	3091.0	1.14	0.117
5	Pack_size + previous_group_type + pack_size:previous_group_type	6	−1539.407	3091.0	1.22	0.112

*Note*: Model 1 best fits the data with the fewest explanatory variables and the lowest AICc. Data were drawn from a subset of wild dogs visiting marking sites and comprised 373 sessions from 11 resident packs across 21 marking sites, which were included as random terms to account for repeated measures.

**TABLE 2:** Effects of the parameters from the most parsimonious model from Table 1.

	Estimate	SE	*z* value	CI (2.5, 97.5%)	Pr (>|z|)
Intercept	1.278	0.249	5.129	0.790, 1.766	2.92e−07***
Pack_size	0.017	0.011	1.591	−0.004, 0.039	.112
Previous_group_size	0.015	0.008	1.813	−0.001, 0.031	.170
Previous_group_type (Stranger)^a^	0.651	0.192	3.391	0.275, 1.027	.0007***
Pack_size:previous_group_type (Stranger)^a^	−0.032	0.012	3.053	−0.053, −0.012	.002**

*Note*: On the factors that may affect the number of scent marks deposited by a pack of wild dogs at a shared marking site. **p < .01; ***p < .001.

^a^ Neighbour was the reference category.

**FIGURE 3:**
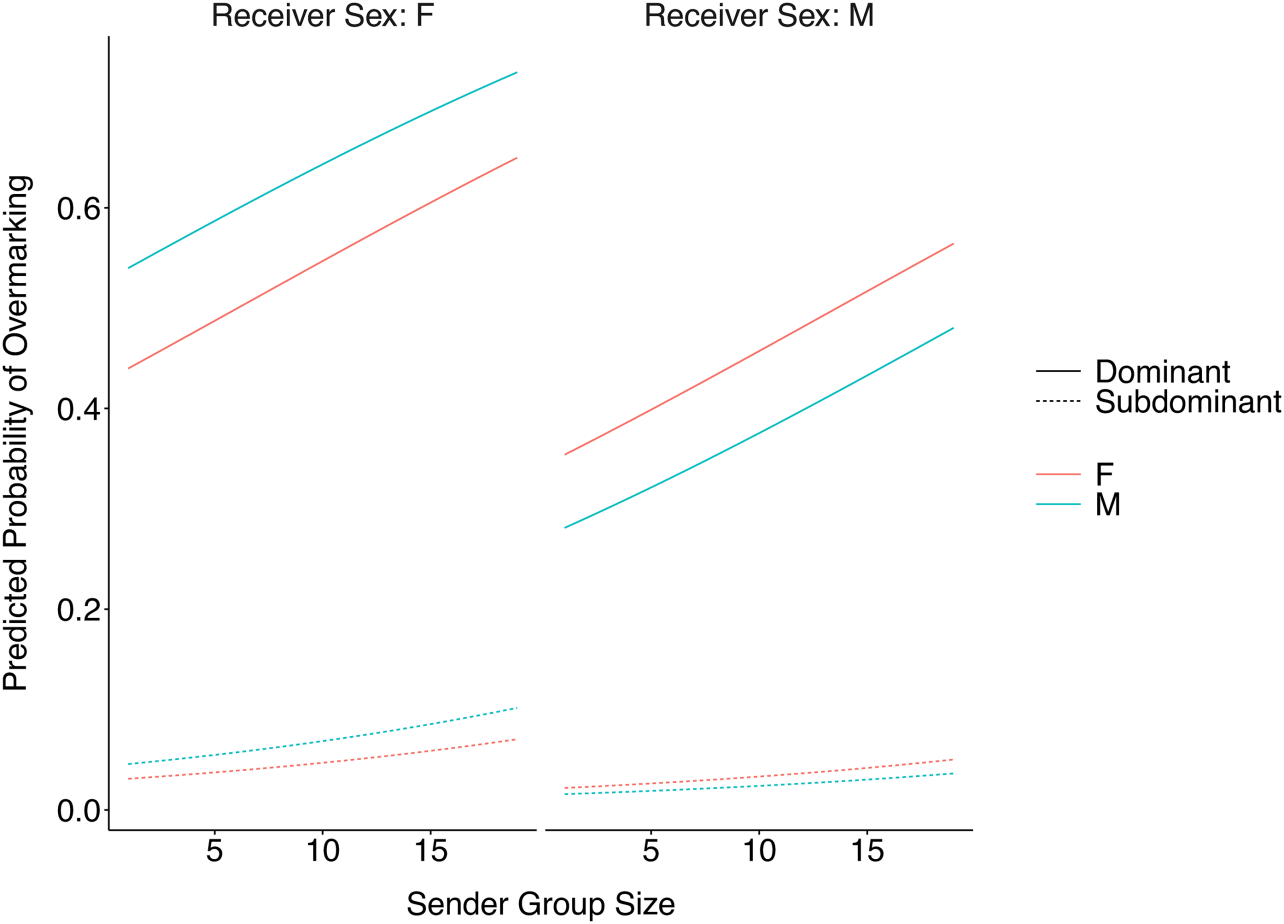
Predicted probabilities of overmarking by wild dogs generated from a GLMM (Table 4) investigating factors that affect the likelihood of overmarking an investigated scent at a shared marking site. For visualisation purposes, we fixed previous group ID to “Neighbour” and season to “non‐mating”. Data were drawn from a subset of wild dogs visiting marking sites, and comprised 2577 sniffing of scent‐mark events across 21 marking sites by 83 individuals across 25 groups.

**FIGURE 4:**
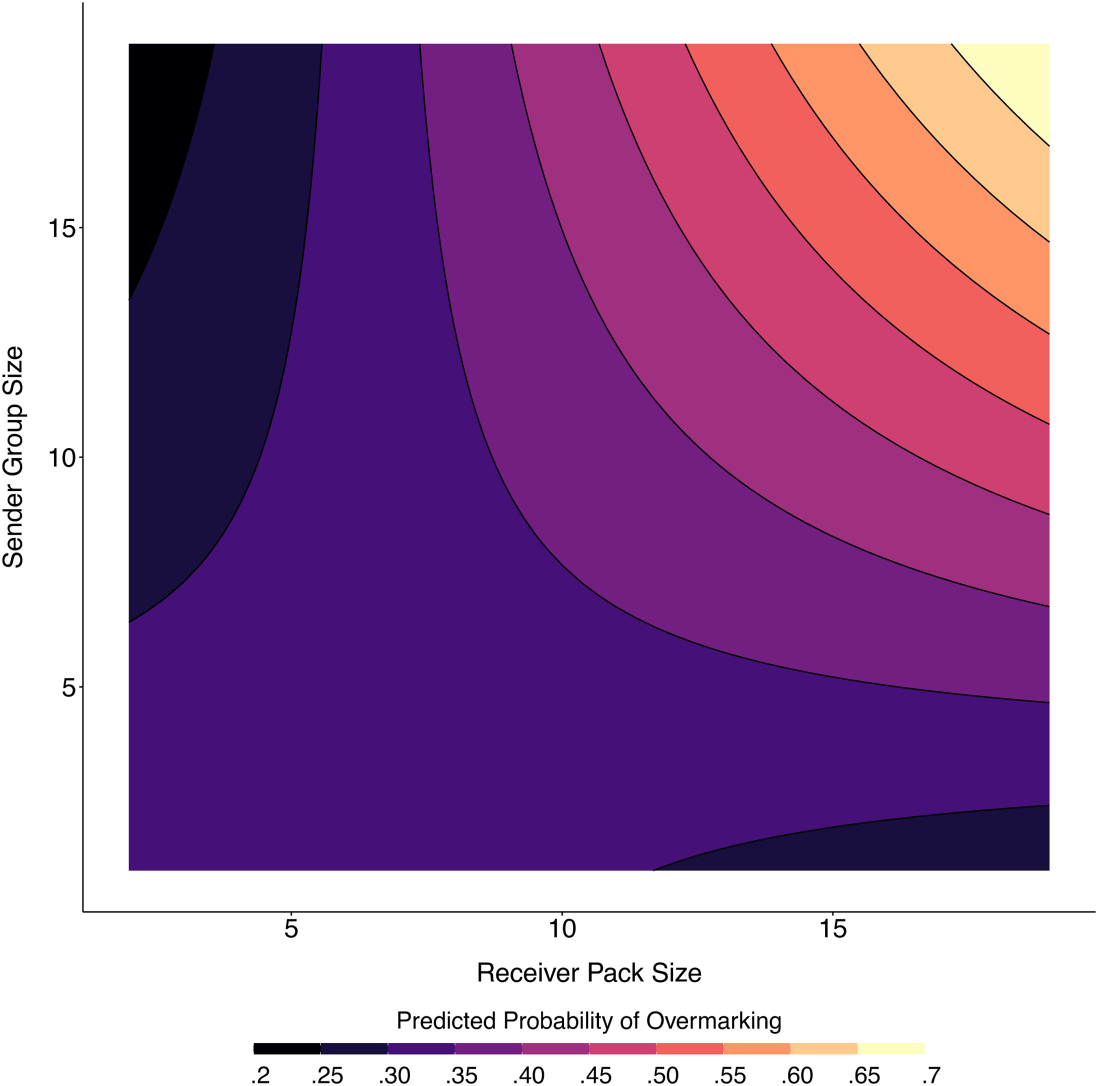
Predicted probabilities of overmarking in wild dogs generated from a GLMM (Table 4) investigating factors that affect the likelihood of overmarking an investigated scent at a shared marking site. For visualisation purposes to explore the interaction between sender group size and receiver pack size, we here fixed sender group type to “Neighbour”, season to “Non‐mating”, the sex of the sender and receiver to “M”, and receiver status to “Dominant”. Data comprised 2577 sniffing of scent‐mark events across 21 marking sites by 83 individuals across 25 groups.

For the second analysis, candidate models were very similar (within <2 AICc of the top performing model), with the top two models having a difference in AICc of <1 (Table 3). The most parsimonious model included the fixed effects sex, status, sender_sex, pack_size, sender_group_size and two interaction terms: pack_size:sender_group_size and sender_sex:sex (Table 4). Overall dominant individuals were significantly more likely to overmark than were subdominants, with dominant females overmarking the most, and subdominant males overmarking the least (Figure 3). However, we found no effect of group type (stranger vs. neighbour) on the marking responses of different demographic classes of wild dog. The interaction between the sex of the sender and the sex of the receiver shows that females are more likely to overmark males and males are more likely to overmark females, regardless of status (Figure 3). There was also a significant interaction between sender pack size and receiver pack size such that individuals from larger packs were more likely to overmark individuals from larger packs than they were to overmark individuals from smaller packs, whereas individuals from smaller packs were less likely to overmark individuals from larger packs than they were individuals from smaller packs (Figure 4).

**TABLE 3:** GLMMs with binomial error distribution exploring how the likelihood of individual overmarking (1 = overmark, 0 = no overmark) of wild dogs at shared marking sites is affected by various factors.

m	Description	df	logLik	AICc	Delta	Weight
Null				1862.6		
1	Sex + status + sender_sex + pack_size + sender_group_size + pack_size:sender_group_size + sender_sex:sex	11	−866.139	1754.4	0.00	0.200
2	Sex + status + sender_sex + pack_size + sender_group_size + season + pack_size:sender_group_size + sender_sex:sex	12	−865.529	1755.2	0.80	0.134
3	Sex + status + sender_sex + pack_size + sender_group_size + sender_group_type + pack_size:sender_group_size + sender_sex:sex	12	−865.982	1756.1	1.71	0.085

*Note*: Model 1 best fits the data with the fewest explanatory variables and the lowest AICc. Data were drawn from a subset of wild dogs visiting marking sites, and comprised of 2577 sniffing of scent mark events across 21 marking sites by 83 individuals from 25 groups, which were included as random terms to account for repeated measures.

**TABLE 4:** Effects of the parameters from the most parsimonious model from Table 3.

	Estimate	SE	*z* value	CI (2.5, 97.5%)	Pr (>|z|)
Intercept	0.082	0.573	0.143	−1.040, 1.204	.886
Sex (M)	−0.361	0.431	−0.838	−1.206, 0.484	.402
Status (Subdominant)^a^	−3.199	0.522	−6.134	−4.222, −2.177	8.55e−10***
Sender_sex (M)^b^	0.414	0.192	2.153	0.037, 0.790	.031*
Pack_size	−0.033	0.032	−1.036	−0.095, 0.029	.300
Sender_group_size	−0.053	0.019	−2.755	−0.089, −0.015	.006**
Pack_size:sender_group_size	0.008	0.001	5.881	0.005, 0.011	4.07e−09***
Sender_sex (M)^b^:sex (M)^b^	−0.738	0.287	−2.576	−1.300, −0.176	.01*

*Note*: On the factors that may affect the likelihood of a wild dog overmarking (1) or not (0) after investigating a scent at a shared marking site. *p < .05; **p < .01; ***p < .001.

^a^ Dominant.

^b^ *F* were the reference categories.

## 4 DISCUSSION

Records from camera trap videos of natural scent marking by African wild dogs at shared latrine sites show that, at the pack level, this species overarchingly exhibits a “dear enemy” behavioural response to competitors, but that this response is dependent on pack size. As the size of the responding pack increases, wild dogs change from reacting more strongly to stranger scent marks (“dear enemy”) to responding more strongly to neighbour scent marks (“nasty neighbour”) (Figure 2). This group level analysis may not fully explain overmarking patterns of wild dogs at marking sites, which may be driven by individual scent marking behaviours. Indeed, at the individual level, and contrary to predictions, the effect of rival type had little effect on overmarking responses, despite sex and social status in part driving patterns of scent marking behaviour overall. That is, dominant females scent marked most, and subdominant males scent marked least, but we found no effect of rival identity on this. Overall, sender and receiver group sizes affect the behavioural responses of individuals, which may underpin apparent dear enemy effects observed at the group level.

Although some species have been considered to conform to either the dear enemy or the nasty neighbour hypotheses (Christensen & Radford, 2018), our results suggest more of a continuum in the behavioural responses of African wild dog packs to rivals' scent, with responses varying according to receiver pack size. Smaller packs respond more strongly to strangers, perhaps because smaller packs are vulnerable to takeovers from similarly sized dispersal coalitions. In contrast, larger packs respond more strongly to neighbours, perhaps because dispersers represent less of a threat, thereby supporting a dear enemy effect. Dholes, another large group‐living social canid, exhibit a similar dear enemy response to rivals overall, but at a finer scale they display different behavioural responses to rival scent than those we describe here (Ghaskadbi et al., 2022). As dhole pack size increases, the intensity of response towards neighbours decreases and increases towards strangers. These differences may reflect different social dynamics, or the small number of dhole packs studied (4 packs studied, range 3–12 individuals; Ghaskadbi et al., 2022). At the group level, given the low AICc difference between the top performing model and the Null model (3 AICc), group behaviour may not best explain observed overmarking patterns of wild dogs at marking sites, with this analysis likely not fully capturing what is driving scent marking behaviour in this species.

Considering individual specific behavioural responses, which may better explain overmarking patterns due to the top performing model being more robust, rival type (stranger or neighbour) may not be the most important social factor driving scent marking, and other characteristics of rivals may play an important role in individual decision making. At this level, group size of both the senders and receivers, as well as social status and sex, appear to drive individual scent marking responses and may underpin the broad scale stranger vs. neighbour responses observed. Individuals from smaller packs were less likely to overmark individuals from larger groups, possibly to avoid detection. Jackson et al. (2012) showed there is a considerable degree of overlap between large packs and small packs territories, with the smaller packs living within the territories of the larger packs. As such, it is possible that small packs may need to change their behaviour to remain cryptic to avoid potentially fatal conflict, although a previous study on this species found no effect of group size on direct encounter outcomes (see, Jordan et al., 2017). Individuals from larger packs were more likely to overmark individuals from large groups, and less likely to overmark individuals from smaller groups (both resident packs and dispersers). This may be because small dispersing coalitions and small packs are of limited threat to large resident packs, while larger packs are often more successful (Woodroffe et al., 2007) and therefore could represent a greater territorial threat. Interestingly, group size does not influence the total number of scent marks deposited during a session, so information regarding sender group size may be attained via other methods such as through memory of direct encounters, at least for known neighbours, given that neighbouring wild dog packs directly encounter one another once every 6 months on average (Creel & Creel, 2002; Jordan et al., 2017; Woodroffe & Donnelly, 2011).

Sex and status influenced overmarking responses in accordance with previous studies on this species in both the inter‐pack (see Claase et al., 2024; Parker, 2010) and intra‐pack context (see Jordan et al., 2014). As in other canids (e.g. Ethiopian wolves, Sillero‐Zubiri & Macdonald, 1998) dominant individuals overmarked more than subdominants, likely because they have the most to lose to intruders. Furthermore, dominant females overmarked more than dominant males in this context, which aligns with results at shared marking sites in this species (see Claase et al., 2024) and in dholes (Ghaskadbi et al., 2022). As dholes exhibit female biased dispersal, this has been interpreted as driving a female‐biased marking pattern (Ghaskadbi et al., 2022), but in African wild dogs both sexes disperse (Behr et al., 2020; McNutt, 1996) suggesting that other factors may be at play in this species. Female‐biased marking in African wild dogs may be driven by uneven resource investment, for example if females are more invested in defending the territory itself, and males are more invested in defending the females holding the territory (see Claase et al., 2024). Contrary to expectation based on the responses of many other species (e.g. meerkats, Mares et al., 2011) wild dogs overmarked individuals of the opposite sex more than they did individuals of the same sex, regardless of their social status. Elevated levels of same sex overmarking are often thought to signal strong competition for resources (Gosling & Roberts, 2001), yet patterns here reflect those seen in the intra‐pack context for dominant individuals (Jordan et al., 2014). Dominants overmarking opposite sexed individuals from different groups may serve to obscure these potential mating opportunities from subdominants within their own pack, thus discouraging dispersal and serving to keep helpers within the pack to assist with pup provisioning. Larger packs raise pups more successfully (Woodroffe et al., 2007), and dominant females, whose investment in offspring is the highest, may be most concerned with discouraging dispersal, which may drive scent marking patterns observed here.

Responses to rivals are likely socially and contextually dependant (Christensen & Radford, 2018; Ghaskadbi et al., 2022) and thus difficult to investigate experimentally. One aspect of scent presentation experimental design which is often oversimplified or held constant for ease of repetition is sender group size (e.g. experiments on banded mongoose, Manser & Müller, 2007; dwarf mongoose, Christensen et al., 2016; meerkats, Mares et al., 2011; dholes, Ghaskadbi et al., 2022; Eurasian beaver, Rosell & Bjørkøyli, 2002; Eurasian badger, Palphramand & White, 2007; African wild dog, Parker, 2010). Here, in our natural experiment, we show that sender group size, and its interaction with receiver group size, may affect behavioural responses, and may drive interpretations of dear enemy or nasty neighbour. Therefore, conclusions from experimental results on species where sender group size has been standardised, or disregarded, must be viewed with caution. Camera trapping of communal latrine sites greatly improves the amount and the breadth of data that can be collected (e.g. sender group size, sender sex and social status), although information of this kind may be more difficult to collect in species where individual identification from camera trap videos is not possible.

In summary, we demonstrate the utility of long‐term camera trapping at communal latrine sites in gathering behavioural observations that can be used in lieu of scent presentation experiments for some species, such as the easily individually identifiable African wild dog. We show that more generally, there are fundamental differences between scent marking patterns resulting from group behaviours and individual behaviours, and that wild dogs exhibit a dear enemy response to intruders overall, but that this response is mediated by pack size and individual overmarking patterns. Smaller packs responded more strongly to strangers while larger packs responded more strongly to neighbours. As such, sender group size – a variable often ignored or held constant in experiments – plays a significant role in determining territorial responses to intruders.

## AUTHOR CONTRIBUTIONS

**Megan J. Claase:** Conceptualization (lead); formal analysis (lead); investigation (lead); methodology (lead); writing – original draft (lead); writing – review and editing (equal). **Mike I. Cherry:** Supervision (equal); writing – review and editing (equal). **J. Weldon McNutt:** Funding acquisition (equal); resources (equal); supervision (equal); writing – review and editing (equal). **Peter J. Apps:** Funding acquisition (equal); resources (equal); writing – review and editing (equal). **Neil R. Jordan:** Supervision (equal); writing – review and editing (equal).

## FUNDING INFORMATION

Funding for this study was generously provided by St Louis Zoo, the Leopardess Foundation and Wild Entrust.

## CONFLICT OF INTEREST STATEMENT

The authors declare no competing interests.

## Supporting information


Appendix S1.

## Data Availability

Data and all R scripts used to process and analyse the dataset are included as Appendix S1 for this article.
